# Red blood cell distribution width/platelet ratio on admission as a predictor for in-hospital mortality in patients with acute myocardial infarction: a retrospective analysis from MIMIC-IV Database

**DOI:** 10.1186/s12871-023-02071-7

**Published:** 2023-04-04

**Authors:** Hongxia Yao, Liyou Lian, Rujie Zheng, Chen Chen

**Affiliations:** 1 Department of Cardiology, the First Affiliated Hospital of Wenzhou Medical University, Wenzhou, China; 2 Department of Radiology, the First Affiliated Hospital of Wenzhou Medical University, Wenzhou, China

**Keywords:** Acute myocardial infarction, Red blood cell distribution width/platelet ratio, MIMIC-IV, Mortality, Prognostic predictor

## Abstract

**Background:**

Red blood cell distribution width (RDW) to platelet ratio (RPR) is a novel inflammatory indicator. It integrates the risk prediction of RDW and platelet, which is associated with adverse outcomes. However, the predictive power of RPR in mortality for patients with acute myocardial infarction (AMI) remains uncertain. Thus, we aimed to explore the association between RPR and 180-day in-hospital mortality in patients with AMI.

**Methods:**

Data on patients with AMI were extracted from the Medical Information Mart for Intensive Care IV (MIMIC-IV) database. Patients were divided into two groups according to the optimal RPR cut-off value. The survival curve between high and low RPR groups was plotted via the Kaplan-Meier (KM) method. Univariate and multivariate Cox regression analyses were performed to determine the association between RPR on admission and 180-day in-hospital mortality.

**Results:**

A total of 1266 patients were enrolled, of which 83 (6.8%) died within 180 days during the hospitalization. Compared with the survivor group, the non-survivor group had higher RPR on admission (0.11 ± 0.07 vs. 0.08 ± 0.06, *P* < 0.001). The KM curve indicated that the survival probability of low RPR group was higher than that of high RPR group. Multivariate Cox regression analysis demonstrated that higher RPR on admission was an independent and effective predictor of 180-day mortality in patients with AMI (hazard ratio [HR]: 2.677, 95% confidence interval [CI]: 1.159–6.188, *P* = 0.021).

**Conclusion:**

Higher RPR was associated with higher in-hospital 180-day mortality in patients with AMI.

## Introduction

With the increasing incidence and prevalence of coronary atherosclerosis, over 200,000 hospital visits each year are associated with myocardial infarction [1, 2]. The acute myocardial infarction (AMI) is characterized by the evidence of myocardial necrosis in a clinical setting consistent with acute myocardial ischemia [3]. Although the diagnostic criteria and major treatment strategies for AMI are well defined [4], the mortality rates for such patients remains high. Therefore, how to define high-risk patients is extremely important for the survival of patients with AMI.

Inflammation is one of the main factors contributing to coronary heart disease which leads to plaque instability and rupture [1]. Red blood cell distribution width (RDW) is associated with heterogeneous cell population [5], which can be used as a reliable indicator for anisocytosis [6]. Recent studies had suggested that inflammation, neurohormone and adrenergic system activation may affect erythrocyte maturation by interfering with erythrocyte membrane, resulting in elevated RDW [5, 7, 8]. RDW has been found to be an extremely effective prognostic predictor in patients with AMI [9, 10].

Platelets play a key role in the pathophysiology of AMI, associated with endothelial cell activation and, together with fibrin, form thrombus within coronary arteries [11]. Platelets not only participate in the formation of blood clots, but also release mediators that form and maintain local inflammatory responses. Several previous studies [12, 13] had shown that a decreased platelet count was associated with the severity of MI, death and reinfarction. However, the relationship between platelet counts and the prognosis of MI was controversial [14–18].

The RDW-to-platelets ratio (RPR), a combination of two independent parameters, has been established as a new index to reflect the severity of inflammation [19, 20]. RPR had been proved as a prognostic marker in breast cancer [19, 21], cardiovascular diseases [22, 23], acute pancreatitis [24, 25] and chronic hepatic [26, 27]. While RPR is related to the clinical prognosis of patients with cardiovascular diseases [22, 23], the predictive power of RPR in mortality for AMI patients remains uncertain. The aim of the present study was to investigate the relationship between RPR on admission and short-term mortality within 180 days in patients with AMI.

## Method

### Study design and patient selection

Data were extracted from the database of Medical Information Mart for Intensive Care IV (MIMIC-IV). The MIMIC-IV database contains clinical data of patients admitted to the Intensive Care Unit (ICU) of Beth Israel Deaconess Medical Center (BIDMC) from 2008 to 2019 inclusive. All patients’ identifiers were removed and patients’ privacy was well protected so that the requirement for informed consent was waived. The MIMIC-IV database is a freely available database, one of the authors who completed the online training course of Data or Specimens Only Research included in Human Research (certification number: 50,765,560) extracted the data. Data extraction was performed using PostgreSQL software (version 6.11).

AMI is defined as being present when blood levels of cardiac troponin (cTn) are increased above the 99th percentile upper reference limit and at least one of the following: 1) Symptoms of MI; 2) New ischaemic electrocardiogram changes; 3) Development of pathological Q waves; 4) Imaging evidence of new loss of viable myocardium or new regional wall motion abnormality in a pattern consistent with an ischaemic aetiology; 5) Identification of a coronary thrombus by angiography or autopsy [28].

Patients who met the diagnosis of AMI were enrolled in the study. Exclusion criteria were as follows: 1) age < 18 years old; 2) patients who were diagnosed with infection, cancer (including hematologic malignancies); 3) RPR on admission records were not available. 4) patients who stayed in the ICU for less than 24 h or without ICU stay record. For patients with multiple admissions, only the first admission data were recorded.

### Data collection

Baseline data including gender, age, ethnicity, and concomitant disease (such as hypertension, diabetes mellitus, cardiac arrhythmia) were extracted. Clinical parameters including Troponin T, Creatine Kinase-MB (CK-MB), blood routine examination and other data were also included in the analysis. The RPR, the main factor we intended to study, was calculated as follows: RPR = RDW / platelet count. We also recorded patients’ surgical procedures including percutaneous coronary intervention (PCI) and coronary artery bypass grafting (CABG). Missing values greater than 30% of parameters were excluded due to potential bias.

### Endpoint event

The endpoint of this study was all-cause in-hospital mortality within 180 days.

### Statistical analyses

The normality of continuous variables was analyzed using the skewness kurtosis normality test. Continuous variables were expressed as the mean with standard deviation (for normal distribution) or median with interquartile range (for non-normal distribution), while categorical variables were presented as numbers and percentages. Independent samples t-tests were performed to compare the differences between the group of survivor and non-survivor group. If continuous variables were normally distributed, otherwise, Mann-Whitney test were used. Chi-square test was used for comparison of categorized variables. Receiver operating characteristic (ROC) curves were used to determine the optimal cut-off value of RPR on admission. Patients in this study were separated into a high admission RPR group and a low admission RPR group according to the optimum cut-off value. The Kaplan-Meier (KM) method was used to plot survival curves of two groups with different RPR on admission, with statistical significance examined by the log-rank test. Univariate and multivariate Cox regression analyses were performed to determine the association between RPR on admission and in-hospital mortality. For variables whose *P* value was less than 0.05 in univariate analysis were considered as potential risk factors and were selected to the multivariate Cox regression model. In addition, the collinearity of variable was tested by variance inflation factors and the equal proportional-hazards assumption also be determined. Interaction analysis was used to determine whether there is an interaction between other variables and RPR. A two-sided *P* < 0.05 was considered statistically significant.

## Results

### Baseline characteristics on enrolled patients

**Fig. 1:**
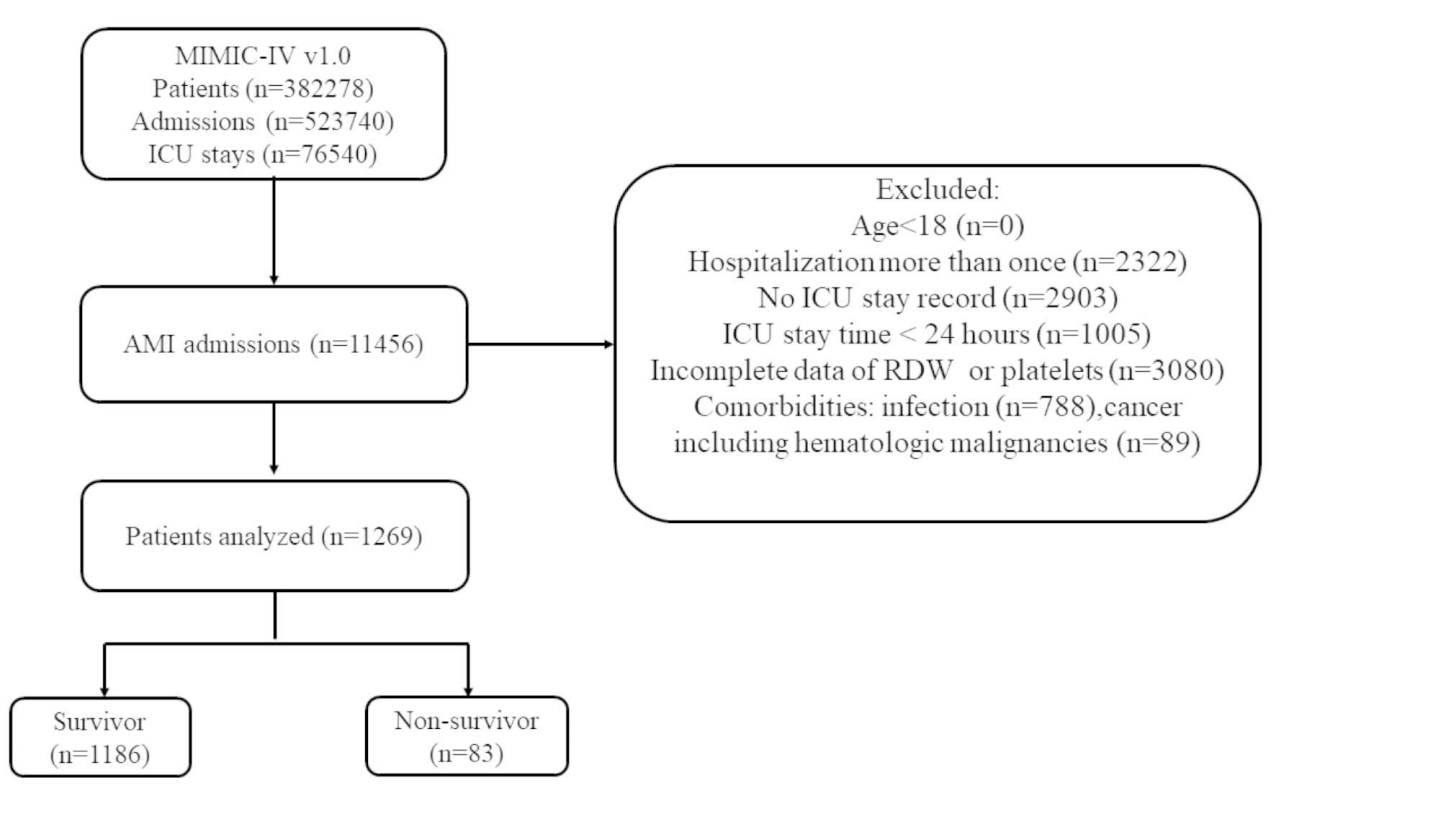
Schematic summary of study and patient selection flow

A total of 1269 patients were enrolled in the study (Fig. 1), of which 843 (66.4%) were men. A comparison of baseline characteristics between the survivor and non-survivor groups based on prognosis within 180 days of admission to ICU was shown in Table 1. The average age of the patients was 68.8 ± 12.6 years. 83 patients died within 180 days of admission (mean length of stay: 122.4 ± 57.9 days). We found that patients in the non-survivor group had higher RPR on admission than patients in the survivor group (0.11 ± 0.07 vs. 0.08 ± 0.06, *P* < 0.001). In addition, other variables such as platelet, RDW, troponin T and creatinine showed significant differences between the two groups. There was no significant difference between the two groups for concomitant disease, such as hypertension, diabetes mellitus, cardiac arrhythmia.

**Table 1:** Baseline characteristics of the study population

Variables	Total	survivor	non-survivor	P-Value
	N = 1269	N = 1186	N = 83	
Men (n, %)	843 (66.4%)	796 (67.1%)	47 (56.6%)	0.050
Age (y)	68.8 ± 12.6	68.5 ± 12.5	73.4 ± 13.0	< 0.001
Ethnicity (white, n, %)	833 (65.6%)	795 (67.0%)	38 (45.8%)	< 0.001
length of hospital stay (days)	122.4 ± 57.9	125.0 ± 57.5	85.3 ± 50.5	< 0.001
Concomitant disease (n, %)				
Hypertension	468 (36.9%)	445 (37.5%)	23 (27.7%)	0.073
Diabetes mellitus	539 (42.5%)	505 (42.6%)	34 (41.0%)	0.773
Cardiac arrhythmia	399 (31.4%)	369 (31.1%)	30 (36.1%)	0.340
Heart failure	641 (50.5%)	597 (50.3%)	44 (53.0%)	0.638
Pulmonary hypertension	76 (6.0%)	73 (6.2%)	3 (3.6%)	0.482
Chronic pulmonary disease	119 (9.4%)	108 (9.1%)	11 (13.3%)	0.210
Peripheral vascular disease	130 (10.2%)	120 (10.1%)	10 (12.0%)	0.575
Cerebrovascular disease	50 (3.9%)	48 (4.0%)	2 (2.0%)	0.653
Renal disease	74 (5.8%)	67 (5.6%)	7 (8.4%)	0.421
Severe liver disease	13 (1.0%)	12 (1.0%)	1 (1.2%)	1.000
Laboratory metrics				
BUN (mg/dL)	26.2 ± 19.2	25.4 ± 18.7	37.9 ± 22.7	< 0.001
Creatinine (mg/dL)	1.5 ± 1.6	1.4 ± 1.5	2.1 ± 1.7	< 0.001
Troponin T (ng/mL)	1.5 ± 3.3	1.4 ± 3.0	2.7 ± 5.6	< 0.001
CKMB (ng/mL)	38.9 ± 72.8	36.6 ± 69.2	68.2 ± 104.9	< 0.001
Glucose (mg/dL)	149.2 ± 73.2	145.4 ± 68.0	204.1 ± 114.3	< 0.001
Sodium (mEq/L)	138.6 ± 4.1	138.7 ± 4.0	137.3 ± 5.3	0.024
Potassium (mEq/L)	4.2 ± 0.6	4.2 ± 0.6	4.4 ± 0.9	0.196
RPR	0.08 ± 0.06	0.08 ± 0.06	0.11 ± 0.07	< 0.001
Lymphocyte (K/ L)	16.9 ± 9.8	17.2 ± 9.7	12.1 ± 9.7	< 0.001
Platelet (K/ L)	216.8 ± 97.3	218.5 ± 97.1	193.0 ± 98.4	0.021
RBC (m/uL)	3.9 ± 0.8	4.0 ± 0.8	3.8 ± 0.8	0.100
RDW (%)	14.6 ± 1.9	14.5 ± 1.8	15.5 ± 2.1	< 0.001
Hemoglobin (g/dL)	11.0 ± 2.3	11.0 ± 2.3	11.0 ± 2.1	0.769
MCH (pg)	30.1 ± 2.3	30.1 ± 2.3	30.2 ± 2.6	0.675
MCHC (%)	33.1 ± 1.5	33.2 ± 1.5	32.6 ± 1.5	0.002
MCV (fL)	90.8 ± 6.2	90.7 ± 6.1	92.7 ± 7.8	0.008
Treatments				
PCI	283 (22.3%)	272 (22.9%)	11 (13.3%)	0.041
CABG	268 (21.1%)	267 (22.5%)	1 (1.2%)	< 0.001

Abbreviation: CK-MB = creatine kinase-MB; BUN = blood urea nitrogen; RPR = red blood cell distribution width/platelet ratio; RBC = red blood cell; RDW = red blood cell distribution width; MCH = mean corpuscular hemoglobin; MCHC = mean corpuscular hemoglobin concentration; MCV = mean corpuscular volume; PCI = percutaneous coronary intervention; CABG = coronary artery bypass grafting

### Clinical outcomes

**Fig. 2:**
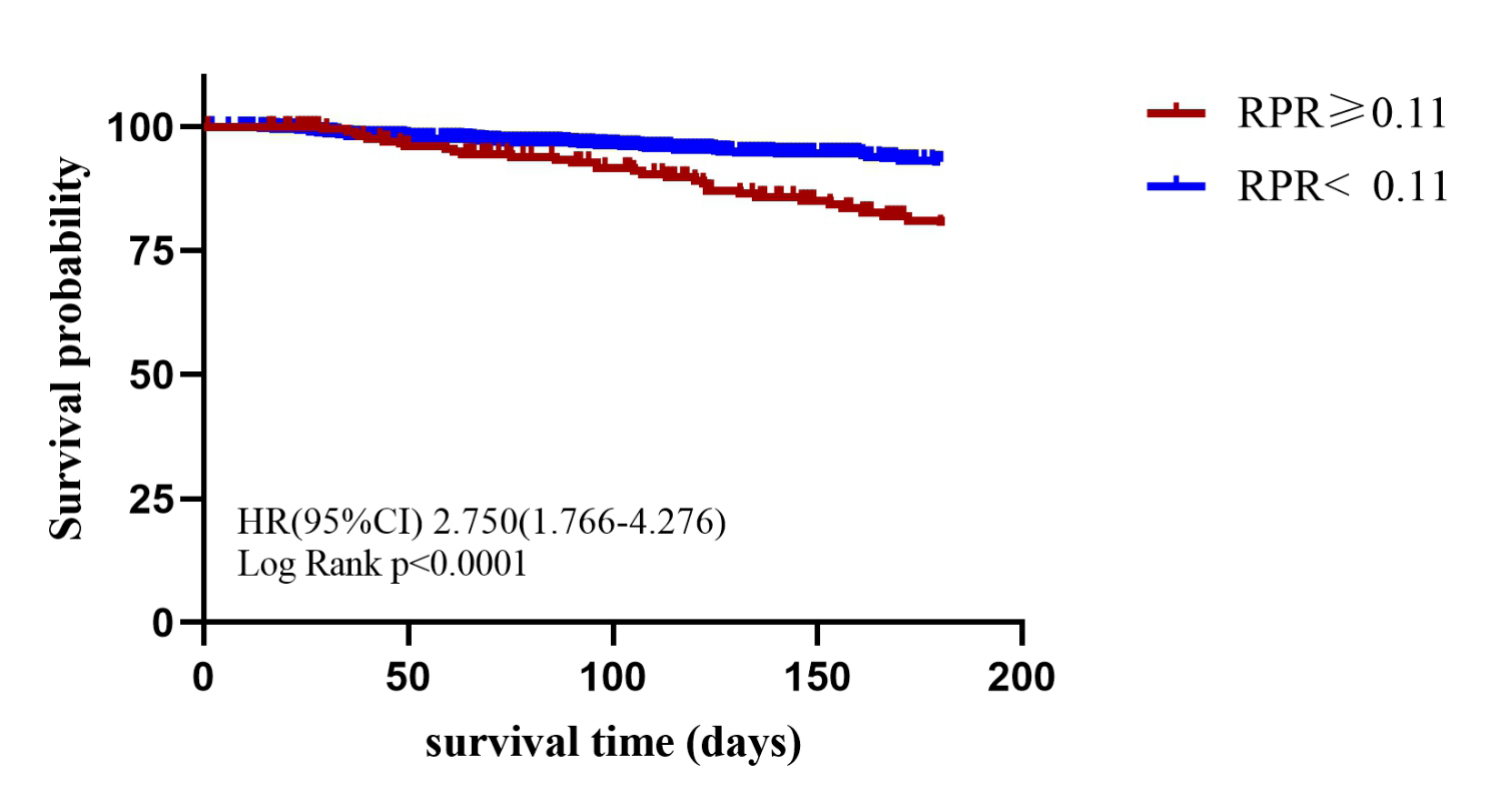
Kaplan–Meier survival analysis for survival probability stratified by RPR

**Fig. 3:**
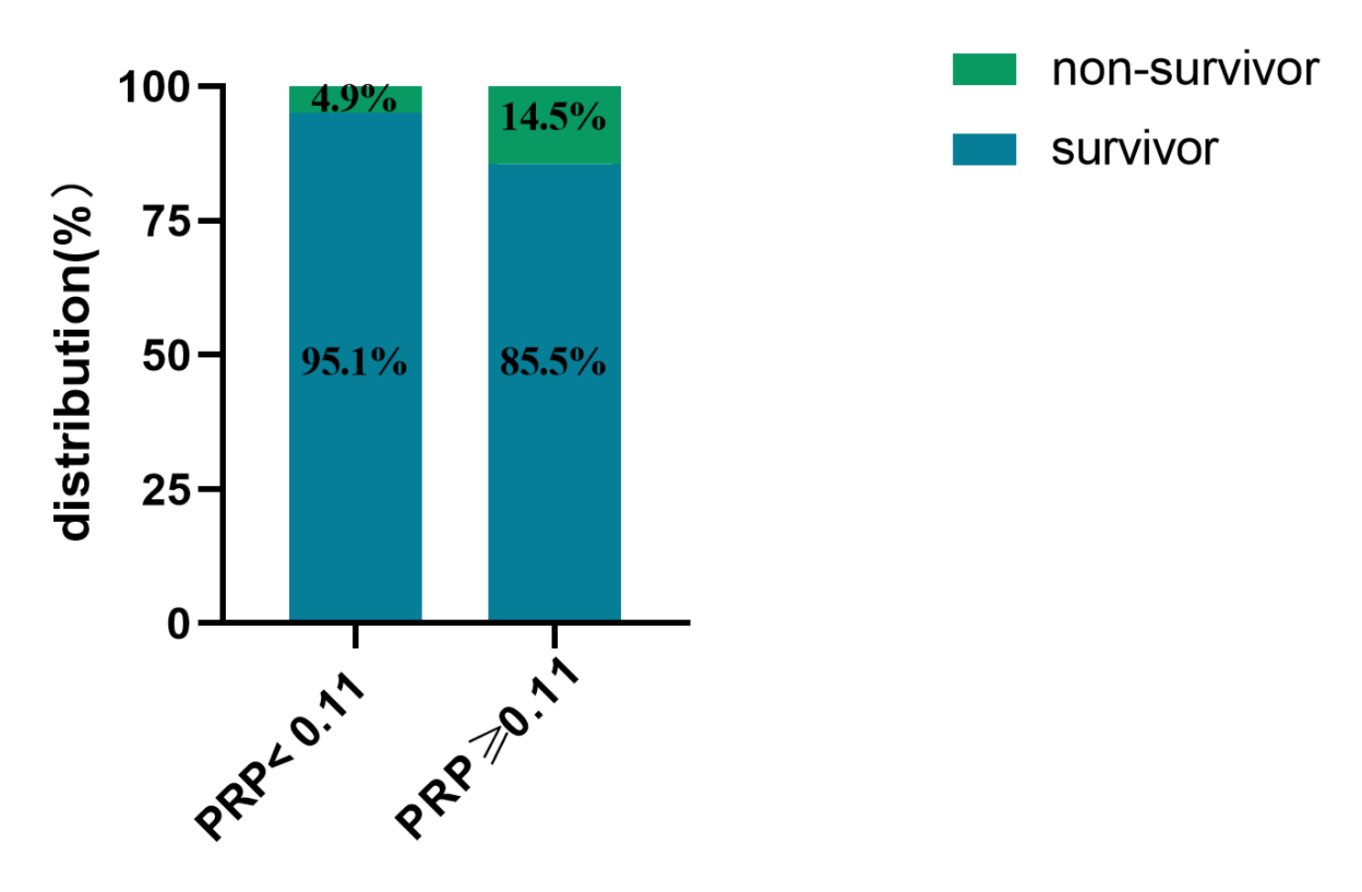
The proportion of mortality of patients with RPR on admission between the high and low groups

The ROC curve showed the optimal cut-off value of RPR on admission was 0.11. There were 221(17.4%) patients with RPR ≥ 0.11 on admission. Figure 2 showed KM curves for in-hospital mortality among patients stratified by RPR on admission. And we found that compared to the low RPR group, the high RPR group had a significantly higher rate of in-hospital mortality (hazard ratio [HR]: 2.750, 95% confidence interval [CI]: 1.766–4.276, *P* < 0.001). Figure 3 showed that the rate of in-hospital mortality of patients in high RPR group was about three times higher than that of patients with low RPR group.

Univariate regression analysis demonstrated that many variables were associated with acute phase mortality within 180 days of patients with AMI (Table 2). Clinical characteristics including gender, age, ethnicity and other factors as well as laboratory parameters such as BUN, creatinine and so on were associated with in-hospital mortality in patient with AMI patients. Interestingly, concomitant diseases such as heart failure, hypertension, and diabetes were not shown to be associated with the endpoint event. Multivariate Cox regression analysis revealed that a higher RPR on admission was an independent risk factor for in-hospital mortality in patients with AMI (HR: 2.677,95% CI: 1.159–6.188, *P* = 0.021), despite confounding factors such as age, ethnicity other factors. We also observed that the older age (HR: 1.031, 95% CI: 1.005–1.507, *P* = 0.019), higher CK-MB (HR: 1.003, 95% CI: 1.000–1.006, *P* = 0.04), and higher blood glucose (HR: 1.004, 95 CI%: 1.002–1.006, *P* < 0.001), were associated with higher acute mortality, while white patients (HR: 0.403, 95% CI: 0.230–0.705, *P* = 0.001) and patients undergoing CABG (HR: 0.084, 95% CI: 0.011–0.623, *P* = 0.015) had a lower risk of death.

**Table 2:** Univariate and multivariate analysis of prognostic variables

Prognostic variables	Univariate analysis		Multivariate analysis	
	HR (95%CIs)	P-value	HR (95%CIs)	P-value
Demographics				
Gender (men)	0.621 (0.401–0.959)	0.032	0.638 (0.368–1.106)	0.109
Age	1.031 (1.011–1.051)	0.002	1.031 (1.005–1.507)	0.019
Ethnicity (white)	0.455 (0.295-0.700)	< 0.001	0.403 (0.230–0.705)	0.001
Concomitant disease (n, %)				
Hypertension	0.796 (0.492–1.288)	0.353		
Diabetes mellitus	0.937 (0.605–1.452)	0.772		
Cardiac arrhythmia	1.136 (0.726–1.778)	0.578		
Heart failure	1.047 (0.680–1.612)	0.834		
Pulmonary hypertension	0.582 (0.184–1.844)	0.358		
Chronic pulmonary disease	1.492 (0.791–2.814)	0.216		
Peripheral vascular disease	1.312 (0.677–2.541)	0.421		
Cerebrovascular disease	0.601 (0.148–2.442)	0.476		
Renal disease	1.440 (0.664–3.123)	0.356		
Severe liver disease	1.413 (0.197–10.155)	0.731		
Laboratory metrics				
BUN (mg/dL)	1.018 (1.011–1.025)	< 0.001	1.001 (0.987–1.015)	0.884
Creatinine (mg/dL)	1.158 (1.071–1.251)	< 0.001	1.141 (0.972–1.340)	0.107
Troponin T (ng/mL)	1.079 (1.033–1.126)	0.001	1.026 (0.961–1.095)	0.449
CKMB (ng/mL)	1.004 (1.002–1.006)	< 0.001	1.003 (1.000-1.006)	0.04
Glucose (mg/dL)	1.005 (1.003–1.006)	< 0.001	1.004 (1.002–1.006)	< 0.001
Sodium (mEq/L)	0.934 (0.894–0.976)	0.002	0.947 (0.893–1.005)	0.075
Potassium (mEq/L)	1.311 (0.970–1.722)	0.078		
RPR (cutoff 0.11)	2.750 (1.766–4.276)	< 0.001	2.677 (1.159–6.188)	0.021
Lymphocyte (K/ L)	0.944 (0.914–0.974)	< 0.001	0.979 (0.949–1.009)	0.169
Platelet (K/ L)	0.997 (0.995-1.000)	0.028	1.001 (0.998–1.005)	0.499
RBC (m/uL)	0.802 (0.602–1.068)	0.131		
RDW (%)	1.181 (1.084–1.287)	< 0.001	0.999 (0.864–1.156)	0.993
Hemoglobin (g/dL)	1.025 (0.922–1.141)	0.645		
MCH (pg)	1.026 (0.934–1.128)	0.589		
MCHC (%)	0.825 (0.718–0.948)	0.007	0.989 (0.811–1.207)	0.915
MCV (fL)	1.047 (1.012–1.085)	0.009	1.037 (0.993–1.084)	0.102
Treatments				
PCI	0.797 (0.421–1.508)	0.485		
CABG	0.036 (0.005–0.256)	0.001	0.084 (0.011–0.623)	0.015

Abbreviation: CK-MB = creatine kinase-MB; BUN = blood urea nitrogen; RPR = red blood cell distribution width/platelet ratio; RBC = red blood cell; RDW = red blood cell distribution width; MCH = mean corpuscular hemoglobin; MCHC = mean corpuscular hemoglobin concentration; MCV = mean corpuscular volume; PCI = percutaneous coronary intervention; CABG = coronary artery bypass grafting

## Discussion

For the first time, the relationship between RPR and mortality in patients with AMI was evaluated. We compared the survivor and non-survivor groups of patients admitted to the ICU with AMI. The main findings of this study are as follows: 1) patients with AMI who died within 180 days had higher RPR on admission; 2) the rate of in-hospital mortality was significantly higher in patients with high RPR than in those with low RPR; 3) RPR on admission was an independent predictor of in-hospital mortality within 180 days in patients with AMI.

Recent studies had shown that elevated RDW was related to higher mortality in patients with AMI [29, 30]. A study of 3100 patients from the MIMIC-IV database showed that a higher RDW was associated with increased risks of in-hospital mortality in patients with AMI [31]. Another study enrolling 763 patients with AMI undergoing a primary PCI proved that a high RDW level on admission was associated with an increased risk for intrahospital cardiovascular mortality [32]. Coronary atherosclerosis is a chronic disease with stable and unstable periods. If the blood vessel wall is irritated by inflammation, a patient who in an unstable period may deteriorate to MI. Increased systemic and local inflammation play a key role in the pathophysiology of Acute coronary syndrome (ACS) [33]. Elevated RDW has been shown to be associated with severe inflammation [34]. Inflammatory factors desensitize bone marrow erythroid progenitor cells, blocking the anti-apoptotic and pro-maturation effects of erythropoietin. Resulting in a large number of reticulocytes to enter the peripheral blood and an increase in RDW [35]. It also led to the change of red blood cells (RBCs) lipid structure, and the destruction of RBCs degeneration ability. As a result, the oxygen carrying capacity of RBCs decreases and the viscosity of whole blood increases, which increases the risk of death in patients with MI [36, 37].

Platelet was associated with the activation of endothelial cells and was important in the pathogenesis of ACS [33]. Several studies had reported the evidence that decreased platelet counts were related to increased MI size and severity and mortality or reinfarction rates [12, 13]. A study demonstrated that decreased platelet counts at hospital admission in post-ACS patients had relation to a significant almost two times higher 5-year mortality rate [38]. Another study including 10,793 patients with ST-segment elevation myocardial infarction (STEMI) from the Thrombolysis In Myocardial Infarction trials database demonstrated that compared with baseline values, a greater decrease in follow-up platelet counts was independently associated with an increased risk of reinfarction at 30 days (OR: 1.44, 95% CI:1.13–1.82, *P* = 0.03) [39] These results were consistent with our findings, that the non-survivor group had lower platelet counts on admission compared with the survivor group.

RPR, as a potential indicator of inflammatory processes, is calculated as the ratio of RDW to platelet count. RPR was considered to be an effective prognostic factor for many diseases [19, 20, 40–43]. One study performed by Lehmann et al. proved that patients after deep-seated intracerebral hemorrhage (ICH) with a low baseline RPR were distinguished by significantly lower case-fatality at 90 days compared to patients with a high baseline RPR (27 vs. 57%; *P* = 0.003, OR: 3.6, 95% CI: 1.6–8.3) [20]. Zhu et al. demonstrated that RPR was an effective predictor of cardiovascular events in hemodialysis patients, with the areas under the ROC curves of 0.88 [41]. Another study including 470 STEMI patients with PCI, demonstrated that higher RPR was associated with an increased risk for in-hospital and long-term major adverse cardiovascular events (MACE), all-cause mortality and cardiovascular mortality [23]. In the development of atherosclerosis, unstable atherosclerotic plaque and formation of clots on the plaque surface, the inflammatory process plays an important role [44]. RPR was a combination of two inflammatory parameters that had been proved have a better ability to predict the prognosis of the disease. A study conducted by Xi et al. confirmed that compared with RDW, RPR presented better diagnostic value of hemophagocytic lymphohistiocytosis (HLH). Moreover, RPR had a better ability to monitor the effects of treatment on HLH [43]. Studies have shown that Troponin T and CKMB are positively correlated with AMI extent [45], which was consistent with our study. In our study, Troponin T and CKMB were both higher in the non-survivor group than in the survivor group, accompanied by higher RPR, suggesting that RPR may also be a predictor of poor outcomes in patients with AMI. RPR, as a readily available and economical blood routine parameter, has been neglected in clinical practice. Our study demonstrated the importance of RPR, as a potential indicator of inflammation, in predicting in-hospital mortality within 180 days in patients with AMI. After adjusting sex, age, concomitant disease, and treatment, RPR remained an independent predictor of mortality in patients with AMI. However, there are few studies on RPR at present. In the current practical application, RPR should be combined with other clinical indicators to better verify the clinical prognosis of AMI.

### Limitations

As a single center, retrospective, small cohort study, there may still be uncontrollable factors affecting the outcome even if adjusted by analysis. We did not conduct subgroup analysis according to the severity of MI, but only analyzed and discussed according to the survival status of patients. A piece of missing data, such as leukocytes and neutrophils, were deleted in this study, these values may affect the outcome. Meanwhile, indicators such as smoking, drinking, and patient medication history, which may have a significant impact on patient outcomes, were not available in the database. The predictive role of RPR for all-cause mortality in patients with AMI in long-term follow-up could not be demonstrated for unavailable follow-up data in the MIMIC-IV database.

## Conclusion

Based on the findings of this study, RPR, as a potential indicator of inflammatory processes, could effectively and independently predict 180-day in-hospital mortality in patients with AMI. More prospective randomized controlled studies are needed for further investigation on the effect of RPR in patients with AMI.

## Data Availability

The data that support the findings of this study are available from MIMIC-IV (Published: March 16th, 2021. Version: 1.0) public database. But restrictions apply to the availability of these data, which were used under license for the current study, and so are not publicly available. Data are however available from the authors upon reasonable request and with permission of the Massachusetts Institute of Technology (Cambridge, MA) and Beth Israel Deaconess Medical Center (Boston, MA).

## List of abbreviations

RDW: Red blood cell distribution width
RPR: RDW to platelet ratio
AMI: Acute myocardial infarction
MIMIC-IV: Medical Information Mart for Intensive Care IV
KM: Kaplan-Meier
HR: hazard ratio
CI: confidence interval
ICU: Intensive Care Unit
BIDMC: Beth Israel Deaconess Medical Center
CK-MB: Creatine Kinase-MB
PCI: percutaneous coronary intervention
CABG: coronary artery bypass grafting
ROC: Receiver operating characteristic
RBCs: red blood cells
ICH: intracerebral hemorrhage
MACE: cardiovascular events
HLH: lymphohistiocytosis
STEMI: ST-segment elevation myocardial infarction
ACS: Acute coronary syndrome
BUN: blood urea nitrogen
MCH: mean corpuscular hemoglobin
MCHC: mean corpuscular hemoglobin concentration
MCV: mean corpuscular volume
cTn: cardiac troponin
